# Healthcare workers’ perceptions of sexual violence during the COVID-19 pandemic in the Eastern Cape

**DOI:** 10.4102/phcfm.v15i1.4087

**Published:** 2023-09-28

**Authors:** Nolundi Kwinana, Charity Masilela, Oladele V. Adeniyi

**Affiliations:** 1 Department of Public Health, Faculty of Health Sciences, University of Fort Hare, East London, South Africa; 2 Department of Biochemistry and Microbiology, Faculty of Sciences, Agriculture and Engineering, University of Zululand, KwaDlangezwa, South Africa; 3 Department of Family Medicine, Faculty of Health Sciences, Walter Sisulu University, Mthatha, South Africa; 4 Department of Family Medicine, Faculty of Health Sciences, Cecilia Makiwane Hospital, East London, South Africa

**Keywords:** children, Eastern Cape, gender-based violence, girls, sexual violence, South Africa, women

## Abstract

**Background:**

The South African government implemented lockdown restrictions in order to prevent the spread of the severe acute respiratory syndrome coronavirus-2 (SARS-CoV-2).

**Aim:**

This study explored the effects of the coronavirus disease 2019 (COVID-19) pandemic on sexual violence in the Eastern Cape province through the lens of healthcare workers’ (HCWs) experiences.

**Setting:**

A Thuthuzela care centre in the Eastern Cape province, South Africa.

**Methods:**

This qualitative study brings together the findings from thematic analysis of semi-structured interviews conducted among 11 purposively selected HCWs in May 2022.

**Results:**

Overall, three themes emerged from the study: the effects of COVID-19 on sexual violence, profile of the survivors and recommendations for combating sexual violence in the region. Most respondents believed that the COVID-19 pandemic caused a surge in the incidence of sexual violence, although all acknowledged that movement restrictions affected reporting. The participants treated mostly black women and children’s survivors, who experienced physical injuries simultaneously. The respondents’ narratives revealed that educational campaigns targeting boys and men could reduce sexual violence in the region. In addition, it was recommended that stricter laws and harsher penalties would serve as deterrents for perpetrators of sexual violence in the country.

**Conclusion:**

The COVID-19 lockdown restrictions exposed the vulnerabilities of black women and children to sexual violence in the study setting. Educational programmes aimed at re-orientating boys and men in both rural and urban communities should be implemented.

**Contributions:**

This study provides an insight into the perceived effect of the COVID-19 pandemic on sexual violence in the Amathole district and South Africa.

## Introduction

Sexual violence remains a public health challenge across the globe and is particularly prevalent in South Africa.^1,2,3^ Sexual violence entails any unwelcomed sexual act that violates the personal dignity of an individual.^4^ Sexual violence can be perpetrated against both males and females; however, statistics indicate that women are predominantly the victims.^4,5^ It has been further demonstrated that approximately one in three women experience physical or sexual violence during their lifetime, mostly by an intimate partner. This constitutes a deplorable violation of women’s and girls’ human rights.^5^

Furthermore, in 2019, prior to the pandemic, an estimated 243 million women and girls aged 15–49 years experienced sexual violence by an intimate partner.^5^ During the coronavirus disease 2019 (COVID-19) pandemic, the problem of sexual violence against women intensified.^6^ Reports from frontline workers indicated that after the COVID-19 pandemic, women and girls experienced a surge in all types of violence.^7,8^ Van der Wath et al.^9^ stated that there are many types of sexual violence; the term encompasses both intimate partner violence (IPV) and non-IPV, with both forms causing physical, emotional and mental harm.

Sexual violence has devastating consequences for survivors, necessitating comprehensive and sustained efforts by multiple stakeholders to end it or at least lower its incidence. During COVID-19 lockdown restrictions, women and children faced several forms of violence as families were confined to homes, with many struggling to survive after losing sources of income.^7,10,11^ The pandemic appears to have intensified women’s and children’s vulnerability; however, estimating the magnitude of sexual violence during COVID-19 remains a daunting task, as many incidents during this time were not reported. Sexual violence affects all socioeconomic strata of the society, but is most prevalent in developing countries where educational levels and socioeconomic status are low, especially in sub-Saharan Africa (SSA).^3,7^

Violence against women and children was rampant in many townships in the Eastern Cape province of South Africa prior to the COVID-19 pandemic.^12^ During the COVID-19 pandemic, several efforts (including the lockdown measures) were directed at preventing the spread of severe acute respiratory syndrome coronavirus-2 (SARS-CoV-2). However, the unintended consequence of the movement restrictions and social confinement unmasked the endemic social and public health crisis of sexual violence in many communities in South Africa.^13^ More so, the inestimable burden of confining people for long periods to often overcrowded dwellings, especially in the rural communities of the Eastern Cape province, have not received researchers’ attention. Given that the Thuthuzela centres across the country offer acute care for the survivors of sexual violence, this study explores the perceived effects of the COVID-19 pandemic on sexual violence through the lens of the frontline healthcare providers in a Thuthuzela Centre in the Eastern Cape province. Findings could potentially inform robust policies aimed at mitigating the impact of disaster event as well as the unintended consequences of strategies to be deployed in every nook and cranny of the country.

## Research methods and design

### Study design and setting

A qualitative phenomenological study was conducted in order to gain an in-depth understanding of the healthcare providers’ perspectives on the perceived effects of the COVID-19 pandemic on sexual violence. The study was conducted in May 2022 at the Thuthuzela Care Centre of Butterworth Hospital in Amathole District of Eastern Cape province, South Africa. This facility is situated in a peri-urban township and provides district healthcare services for the residents, serving as a referral centre for several community health centres in the rural areas surrounding Butterworth. The Thuthuzela Centre is a designated facility for acute and follow-up care of survivors of sexual violence in the region. The facility is manned by various categories of healthcare providers: doctors, forensic and general professional nurses, social workers, counsellors and law enforcement agents (police). They provide acute care, debriefing, counselling, post-exposure care packages and evidence collection for forensic examination.

### Participants

Participants comprised a multidisciplinary team of healthcare providers (professional nurses, social workers, counsellors and police officers) who were directly involved in providing acute care for survivors of sexual violence in the hospital. A total of 11 key informants were purposively selected using convenient sampling and interviewed from the study setting. Participants were selected if they had worked in the centre for at least 1 year and were available during the period of the study. The purpose and process of the study was explained to them and those who agreed to participate signed written informed consent forms prior to being interviewed.

### Data collection procedure

A trained interviewer conducted semi-structured interviews, adopting an open-ended technique to elicit in-depth information on participants’ perspectives on the effects of COVID-19 lockdowns on sexual violence in the area. Key informant interviews lasted an average of 40 min each. Prior to the interviews, a pilot study was conducted with three nurses to assess the validity of the interview guide. Following a review of results of the pilot study, a valid interview guide was designed and used for the main study. The interview guide comprised three main questions:

- What are the effects of COVID-19 on sexual violence in the region?
- Who are the clients managed in the Thuthuzela centre during the COVID-19 pandemic?
- What are your recommendations on how to address the problem of sexual violence in this region?

When necessary, the interviewer used probing techniques to elicit additional information so that data yield was comprehensive and sufficiently deep.

Interviews were conducted in English because all the healthcare providers had used this language as the medium of their training. Interviews were audiotaped and the interviewer also kept field notes of the process. Each participant was asked to introduce herself; eliciting age, professional category, and years of working with survivors of sexual violence. In addition, the interviewer asked open-ended questions about the effects of COVID-19 lockdowns on sexual violence and the profile of survivors. Perspective of the participants on how to combat this widespread social ill in the region was further elicited. Recruitment of key informants continued until no new information emerged during the interviews, affirming that point of data saturation has been reached.

### Data analysis

The qualitative data obtained from key informant interviews was transcribed verbatim and coded, with additional information provided by the field notes. Thematic content analysis was then conducted on the transcriptions. All transcriptions and field notes were read several times and cross-checked to ensure consistency and accuracy. Responses were categorised into themes, with all themes colour-coded for ease of reference.

This process ensured that all relevant information, including unanticipated comments, were grouped and coded appropriately. Thematic analysis was carried out concurrently with data collection to identify the point of saturation, which was achieved after the 11th interview. A critical and reflexive review of the transcripts and field notes revealed a narrative of how the healthcare providers perceived the effects of COVID-19 on sexual violence and their recommendations for mitigating this social ill in the region. To ensure data credibility, four interviews (audiotapes, field notes and coding of themes) were selected randomly and reviewed independently by all three authors. This ensured that there was agreement on the accuracy of the transcriptions and the coding of themes before the analysis was completed.

### Ethical considerations

The University of Fort Hare Ethics Committee granted approval for the study’s implementation (20211002KwinanaFBN). All key informants gave their written informed consent to participate as volunteers in the study. Participants were guaranteed confidentiality and anonymity. The study was conducted in line with the Helsinki Declaration and good clinical practice guidelines.

## Results

Table 1 shows the characteristics of the participants. The data show that a multidisciplinary team of stakeholders was involved in responding to gender-based violence in the region, comprising professional nurses, counsellors, police officers and social workers. The ages of the participants ranged from 34 to 58 years, while their years of work experience ranged from 3 to 10 years, with a mean of 5.9 years.

**TABLE 1:** Characteristics of the key informant participants.

Participant code	Age	Gender	Profession	Years of work experience
1. P1	58	Female	Professional nurse	9
2. P2	45	Female	Professional nurse	4
3. P3	37	Female	Professional nurse	3
4. P4	34	Female	Professional nurse	4
5. C1	49	Female	Counsellor	8
6. C2	38	Female	Counsellor	5
7. C3	41	Female	Counsellor	6
8. PO 1	35	Female	Police officer	3
9. PO 2	39	Female	Police officer	5
10. SW 1	52	Female	Social worker	10
11. SW 2	42	Female	Social worker	8

P, professional nurses; C, counsellors; PO, police; SW, social worker.

### Themes

Three themes emerged from the key informant interviews:

- the effects of COVID-19 on sexual violence
- the profile of the survivors of sexual violence
- recommendations for combatting sexual violence in the region.

#### Theme 1: Effects of COVID-19 on sexual violence

Most of the participants believed that lockdowns forcing people to remain in their homes resulted in an increase in conflicts among couples and families. Several commented that since people were accustomed to going to work, or at least leaving their homes during the daytime, confinement to the home caused frustration which expressed itself in angry outbursts and violence. A participant expressed this view in the following words:

‘It was unusual for people to stay in the same place for such a long time without going to work, so it affected people in such a way that people had more quarrels with their lovers and boyfriends.’ (P2, 45 years old, four years’ work experience).

Some participants highlighted the fact that because of restrictions to movement, fewer cases of sexual violence were reported during the pandemic than before:

‘It’s the reports that decreased, because there was an issue of transport and the time allocated for moving of vehicles. People who came are those that are close, and those that called the police or saw the police van and ran for help. Then they came with the police van. I would say it’s the reports that decreased, otherwise rape got worse within closed doors by close relatives.’ (P4, 34 years old, four years’ work experience).

Two participants (P2 and C2) made the case that in their view, sexual violence increased during COVID-19, but people were not able to report such cases because of restrictions:

‘There was a difference because before COVID-19, cases of assaults and sexual violence were worse, but during COVID-19 there were few cases reported. The statistics were high during December, because it’s the festive season and people tend to celebrate or go to social gatherings in December.’ (P2, 45 years old, four years’ work experience; C2, 38 years old, five years’ work experience).

One or two felt that the lower number of reported cases may have had to do with lowered consumption of alcohol during this time because alcohol retailers were forced to close during lockdowns.

#### Theme 2: Profile of the survivors of sexual violence

All the participants noticed that the survivors they dealt with were mostly black African females. According to P3, young children and young adults were more frequently sexually assaulted than women in other age groups. PO1 explained that children, in particular, were victims of sexual assault:

‘If it’s a child, it’s rare to get physical assault, but mostly its sexual assault.’ (PO1, 35 years old, three years’ work experience).

PO1 further observed that sexual assault was more likely to occur in younger females, while physical assault was more common among older females. P4 added to this:

‘Physical assault is rare … sexual violence is more common, and most people who experience or go through sexual assault are threatened, so they give in easily to the abuser without being physically assaulted.’ (P4, 34 years old, four years’ work experience).

This explanation provided by P4 was reinforced by SW1 who spoke of a young girl who had been raped:

‘She says that her uncle started raping her when she was eight years, and when the uncle is raping her, he takes out a gun and also a knife. She also said that he even cut himself to scare her not to report him. What worries me is that the uncle is a jailbird. He was once beaten by the community and is not scared of jail. The girl is 13 years now.’ (SW1, 62 years old, ten years’ work experience).

#### Theme 3: Recommendations on how to combat sexual violence in the region

All participants felt strongly that more needed to be done to combat sexual violence in the country. The most frequent suggestion was an intensification of educational campaigns and initiatives. P4 advocated more campaigns in both rural and urban areas with a focus on boys and men:

‘Firstly, most perpetrators of sexual violence like to say they grew up in an abusive situation. So, there should be classes where our boy child will be taught respect, love, help and sharing. When it’s like that, they will easily notice something wrong here. That’s when social workers will have to be involved.’ (P4, 34 years old, four years’ work experience).

The participants further advocated stricter laws and sentences for perpetrators. P3 said that perpetrators need to know that there are serious consequences for sexual violence and that fear of the consequences should deter them from committing rape. In addition, for survivors to report cases of sexual violence, they need to know that their perpetrators will not come back and hurt them:

‘If there could be harsher sentences … because survivors feel afraid and unprotected, because when some come and report the cases of rape, they are afraid to go back home since the perpetrator is roaming the street and she sees that this person can still come back for her.’ (P3, 37 years old, three years’ work experience).

‘Creating a safe environment for children. We can all be voices of sexual violence wherever we are. It starts with you to stand up to your friend that you know is abusing someone. We can’t wait for health workers only. Let us all unite against this evil act. We need to speak in one language and say “no” to sexual violence. Even if you know the perpetrator, stand up for the survivors.’ (P3, 37 years old, three years’ work experience; C3, 41 years old, six years’ work experience; SW2, 42 years old, eight years’ work experience).

The responses by the participants indicate the strong feelings held by those at the frontline of treating cases of sexual violence. These healthcare workers deal with appalling cases of sexual violence to women and children daily, and their recommendations ought to be noticed and acted on. The point made that all in society have a role to play in confronting the scourge of sexual violence is an apt one. Given the high incidence of sexual violence, it is inevitable that many people know someone who is a perpetrator. A combination of educational campaigns – especially among boys – standing up to perpetrators and harsher sentences for perpetrators is likely to help bring down the high rate of sexual violence in South Africa.

## Discussion

This study provides insights into the perceptions of healthcare workers on the effects of the COVID-19 pandemic on sexual violence in the Eastern Cape. The findings reveal that the incidence of sexual violence was high in the sampled area before the COVID-19 pandemic and that reported findings dropped during lockdown restrictions. However, the drop was attributed largely to lockdown restrictions on movement and difficulty of reporting, rather than to a reduced incidence of sexual violence.

It was clear from the participants’ responses that communities such as the one surrounding the Thuthuzela Centre in Butterworth Hospital struggled with lockdown conditions, mostly because of loss of income. This, coupled with the harsh penalties for being found in public places during lockdown, frustrated people, causing tensions in households. This finding supports the assertion made by Aziz and Moussa et al.^5^ that policymakers did not consider vulnerable individuals when the COVID-19 interventions were designed. The lockdown strategy served one purpose – curbing the spread of the virus – but had the unintended consequence of exacerbating sexual violence, largely because of the heightened frustration experienced by men.^8^ At the same time, this study highlighted the possibility that sexual violence figures may have been lower than expected during this time owing to an enforced reduction in the use of alcohol. Alcohol consumption has been shown to be a catalyst for physical and sexual violence.^14,15^ It has been shown elsewhere that the alcohol ban during lockdowns prevented some of the short-term effects of alcohol consumption.^16,17^

The study’s findings show that restrictions of movement during the COVID-19 pandemic prevented survivors of sexual violence from reporting their abuse to the authorities, with most having to endure it in isolation. This research corroborates the findings of Kaswa et al.^18^ who demonstrated that the COVID-19 pandemic had a trickle-down effect on vulnerable members of the society, with violence against women increasing even though fewer cases were reported in many areas. Other studies have shown that the public health measures deployed by governments to contain the spread of SARS-CoV-2 such as lockdowns, quarantines, social distancing, travel bans and shutdowns of non-essential services caused socioeconomic disruption and disproportionately affected vulnerable populations.^19,20^

The study’s findings also show that young girls were more susceptible to sexual violence than any other age group, while older women were more commonly victims of physical assault. In addition, the findings suggest that black women of African descent are more likely to experience violence than any other ethnic group in South Africa. Similarly, Crooks et al.^8^ found that African American women were more likely to be victims of violence than women of other race groups in the USA. The study’s findings show that black women continue to bear the burden of all forms of violence, with COVID-19 restrictions exacerbating conditions that already existed in this cohort.^8^ The findings also show that girls under the age of 18 are especially vulnerable to sexual violence, largely from people they know, including family members. This resonates with observations made by Gewirtz-Meydan and Finkelhor in 2020, who stated that children and young adults are particularly exposed to sexual violence from people they know, including relatives and neighbours.^11^

Governments should view sexual violence as a social and public health disaster that requires aggressive action to combat and prevent, particularly in South Africa, where women and girls remain susceptible to all forms of abuse and exploitation. The recommendation that men and boys be targeted for educational campaigns ought to be taken up with vigour, with programmes implemented in both rural and urban areas. These programmes should be multipronged in nature and offered by multiple stakeholders, to ensure comprehensive coverage of the many issues that contribute to sexual violence against the vulnerable population in the society. As suggested by Isike et al.,^21^ the concept of re-socialising boys and men for positive gender relations could be a pragmatic solution to this public health crisis in the country. In addition, Chauke et al.^7^ pointed out that youth workers should be equipped with relevant skills to empower women so that they can help prevent violent acts in their surroundings.^7^

Participants in this study made a strong plea for authorities to enact and institute stricter laws and harsher sentences for perpetrators of sexual violence. The problem of sexual violence is a societal one that requires every stakeholder to be involved in combatting it.^6^ Among others, it should be observed that sexual violence is an outright violation of the constitutional rights of women and children, which is protected by the constitution (*Act no. 108 of 1996*) and Bill of Rights.^22^ The rights of women and other vulnerable populations against all forms of violence affirm the democratic values of human dignity, equality and freedom, which must be protected by the relevant authorities. This position was reiterated through the declaration by President Cyril Ramaphosa on the 28 January of 2022 by strengthening the laws to protect women and children against sexual violence and other forms of gender-based violence. The president also signed the following legislations into law; the Criminal Law (Sexual Offences and Related Matters) Amendment Act Bill, the Criminal and Related Matters Amendment Bill and the Domestic Violence Amendment Bill as commitment to do the needful to protect women’s rights. The pragmatic changes in the existing laws are aimed to further mitigate the increasing trends of sexual violence (both intimate and non-intimate violence) in the country. In addition, interventions against sexual violence in the country should be holistic and robust to ensure the safeguarding of vulnerable populations.

### Limitations of the study

Given that this was a single centre study with a qualitative design, findings are not generalisable to the rest of the country. However, it is believed that the perceptions of professionals who deal with the survivors reflect the broader views of other healthcare providers in similar settings in the country. A quantitative study aimed at estimating the effect of COVID-19 pandemic on the incidence of sexual violence across the country should follow to validate some of the views expressed by the participants.

## Conclusion

The COVID-19 lockdown restrictions exacerbated the vulnerabilities of black women and children in the study region to sexual violence, especially sexual violence. A drop in reported cases of sexual violence during the COVID-19 pandemic was ascribed mostly to restrictions on movement during lockdown, which made reporting difficult. It is imperative that programmes aimed at re-orientating boys and men in both rural and urban communities be implemented to reduce the incidence of sexual violence in the region. In addition, government should enact and enforce stricter laws to mitigate sexual violence in the country. Future disaster plans should be cognisant of unintended consequences, and integrate measures to mitigate the escalation of sexual violence in the country during future disasters or emergency situations.

## References

[1] Ajayi AI, Mudefi E, Owolabi EO. Prevalence and correlates of sexual violence among adolescent girls and young women: Findings from a cross-sectional study in a South African university. BMC Women’s Health. 2021;21(1):299. 10.1186/s12905-021-01445-8 34399751 PMC8365970

[2] Perrin N, Marsh M, Clough A, et al. Social norms and beliefs about gender based violence scale: A measure for use with gender based violence prevention programs in low-resource and humanitarian settings. Confl Health. 2019;13(1):6. 10.1186/s13031-019-0189-x 30899324 PMC6408811

[3] Ahmed SAKS, Ajisola M, Azeem K, et al. Impact of the societal response to COVID-19 on access to healthcare for non-COVID-19 health issues in slum communities of Bangladesh, Kenya, Nigeria and Pakistan: Results of pre-COVID and COVID-19 lockdown stakeholder engagements. BMJ Glob Health. 2020;5(8):e003042. 10.1136/bmjgh-2020-003042 PMC7443197 32819917

[4] Anyanwu JC, Salami AO. The impact of COVID-19 on African economies: An introduction. Afr Dev Rev. 2021;33(S1):S1–S16. 10.1111/1467-8268.12531 34149237 PMC8207010

[5] Aziz ZA, Moussa J. COVID-19 and violence against women: Unprecedented impacts and suggestions for mitigation. In: Kjaerum M, Davis MF, Lyons A, editors. COVID-19 and Human Rights. Routledge; 2021.

[6] Bradbury-Jones C, Isham L. The pandemic paradox: The consequences of COVID-19 on domestic violence. J Clin Nurs. 2020;29(13–14):2047–2049. 10.1111/jocn.15296 32281158 PMC7262164

[7] Chauke TA. Understanding Gender-Based Violence prevention among young women : Youth workers perspective?. Afr J Gend Soc Dev. 2021;10(1):173–191. 10.31920/2634-3622/2021/v10n1a8

[8] Crooks N, King B, Tluczek A, Sales JM. The process of becoming a sexual black woman: A grounded theory study. Perspect Sex Reprod Health. 2019;51(1):17–25. 10.1363/psrh.12085 30650233 PMC7998511

[9] Van der Wath A. Women exposed to intimate partner violence: A foucauldian discourse analysis of South African emergency nurses’ perceptions. Afr Health Sci. 2019;19(2):1849–1857. 10.4314/ahs.v19i2.7 31656467 PMC6794526

[10] Fouché A, Truter E, Fouché DF. Safeguarding children in South African townships against child sexual abuse: The voices of our children. Child Abus Rev. 2019;28(6):455–472. 10.1002/car.2603

[11] Gewirtz-Meydan A, Finkelhor D. Sexual abuse and assault in a large national sample of children and adolescents. Child Maltreat. 2020;25(2):203–214. 10.1177/1077559519873975 31526040

[12] Hendricks EA. The effects of the exposure to violence in schools on the psychological well-being of learners in the Sarah Baartman District Municipality, Eastern Cape. Afr J Soc Work. 2019;9(2):1–9.

[13] Mahlangu P, Gibbs A, Shai N, Machisa M, Nunze N, Sikweyiya Y. Impact of COVID-19 lockdown and link to women and children’s experiences of violence in the home in South Africa. BMC Public Health. 2022;22(1):1029. 10.1186/s12889-022-13422-3 35597933 PMC9123923

[14] Sontate KV, Rahim Kamaluddin M, Naina Mohamed I, et al. Alcohol, aggression, and violence: From public health to neuroscience. Front Psychol. 2021;12:699726. 10.3389/fpsyg.2021.699726 35002823 PMC8729263

[15] Mouilso ER, Wilson LF. Alcohol and sexual assault. In: Handbook of sexual assault and sexual assault prevention. Cham, Switzerland: Springer Nature Switzerland AG; 2019. p. 195–209.

[16] Jamieson L, Mathews S, Röhrs S. Stopping family violence: Integrated approaches to address violence against women and children. Child Fam State. 2018;1:81–92.

[17] Theron M, Swart R, Londani M, Parry C, Petersen Williams P, Harker N. Did COVID-19-related alcohol sales restrictions reduce alcohol consumption? Findings from a National Online Survey in South Africa. Int J Environ Res Public Health. 2022;19(4):2422. 10.3390/ijerph19042422 35206607 PMC8878112

[18] Kaswa R. The impact of the COVID-19 pandemic on healthcare service access for the victims of sexual assault. S Afr Fam Pract (2004). 2021;63(1):5367. 10.4102/safp.v63i1.5367 PMC8602966 34797091

[19] Meel B. Profile of victims of rape in the Transkei sub-region of South Africa (2006–2014). Indian J Forensic Med Toxicol. 2020;14(4):632–636.

[20] Muluneh MD, Stulz V, Francis L, Agho K. Gender based violence against women in Sub-Saharan Africa: A systematic review and meta-analysis of cross-sectional studies. Int J Environ Res Public Health. 2020;17(3):903. 10.3390/ijerph17030903 32024080 PMC7037605

[21] Isike C. The value of re-socializing boys and men for positive gender relations to curb gender-based violence and femicide in South Africa. In: Fontaine-Skronski K, Thool V, Eschborn N, editors. Does the UN model still work? Challenges and prospects for the future of multilateralism. Brill, 2022; p. 266–286.

[22] Constitution SA. Chapter 2: Bill of Rights. Pretoria: The constitution of the Republic of South Africa, 1996; p. 6–24.

